# Severe erytrodermic psoriasis in child twins: from clinical-pathological diagnosis to treatment of choice through genetic analyses: two case reports

**DOI:** 10.1186/1756-0500-7-929

**Published:** 2014-12-17

**Authors:** Elena Campione, Laura Diluvio, Alessandro Terrinoni, Augusto Orlandi, Maria Paola Latino, Claudia Torti, Lucia Pietroleonardo, Elisabetta Botti, Sergio Chimenti, Luca Bianchi

**Affiliations:** Department of Dermatology, Tor Vergata University of Rome, Rome, Italy; Department of Experimental Medicine, IDI-IRCSS, Tor Vergata University of Rome, Rome, Italy; Department of Anatomic Pathology, Tor Vergata University of Rome, Rome, Italy

**Keywords:** Erythroderma, Psoriasis, Infancy, Cyclosporine

## Abstract

**Background:**

Pediatric erythroderma is a severe cutaneous disorder, which may pose diagnostic and therapeutic challenges. Psoriasis, ichthyoses, atopy, seborrhoeic dermatitis, pityriasis rubra pilaris, infections, metabolic diseases, drugs reaction, may cause erythroderma. The therapy should be tailored on each aetiology, if possible. The biochemical and metabolic imbalance should be corrected, and particular attention should be paid to the psychosocial behavior often related to this disfiguring disease.

**Case presentation:**

Two 3 year-old Caucasian twins have been suffering from an unmanageable erythroderma since the age of 8 months. The diagnosis of psoriasis, already remarkably expressed in the father’s family in three cases of fraternal twins, could be enforced for several points. Major histocompatibility complex, class I, Cw*06 was detected in both twins; we found no transglutaminase-1, no corneodesmosin, nor any Interleukin-36 receptor antagonist gene mutations. We performed a cutaneous histology, positive immunostaining for Lympho-epithelial Kazal-type-related inhibitor, dermoscopy and reflectance confocal microscopy. The twins had previously received systemic steroids, short cycles of low-dosage ciclosporine, followed by etanercept at the dosage of 0,8 mg/kg, without reliable results. Cyclosporine was then reconsidered at a dosage of 5 mg/kg/day with close blood monitoring. After three months of treatment, consistent clearing and significant improvement of their social and psychological behaviour were achieved. After over one year of continuous therapy with cyclosporine, the twins have still maintained the result obtained.

**Conclusion:**

Pediatric erythroderma may pose a great challenge as a potentially life-threatening condition causing extreme distress in children, parents and pediatricians. In young patients it is mandatory to establish correct clinical and instrumental procedures, possibly supplemented by genetic analyses such as those we required, in order to determine an effective and safe therapy in terms of cost-benefit and put patients and family in the best condition to perform common daily activities.

## Background

Children erythroderma may be due to several not related, inherited or acquired, cutaneous disorders such as psoriasis, ichthyoses, pityriasis rubra pilaris, atopy, seborrhoeic dermatitis, staphylococcal-scalded skin syndrome, infections, metabolic diseases, drug-induced or unidentified forms [1–3]. Erythroderma is a severe skin disease - even a possible cause of medical emergency – and a potentially life threatening disorder, which may offer problems of misdiagnoses or mismanagements [4–7]. The therapy should be tailored on each different aetiology, if possible, and supported by correcting the haematological, biochemical and metabolic imbalance. Moreover, particular attention should be paid to the psychosocial behavior often related to this chronic disfiguring skin condition [8]. Herein, we report how a continuous cyclosporine regimen could be an effective and safe treatment for severe psoriatic erythroderma in two three-year-old Caucasian twins.

## Case presentation

**Figure 1:**
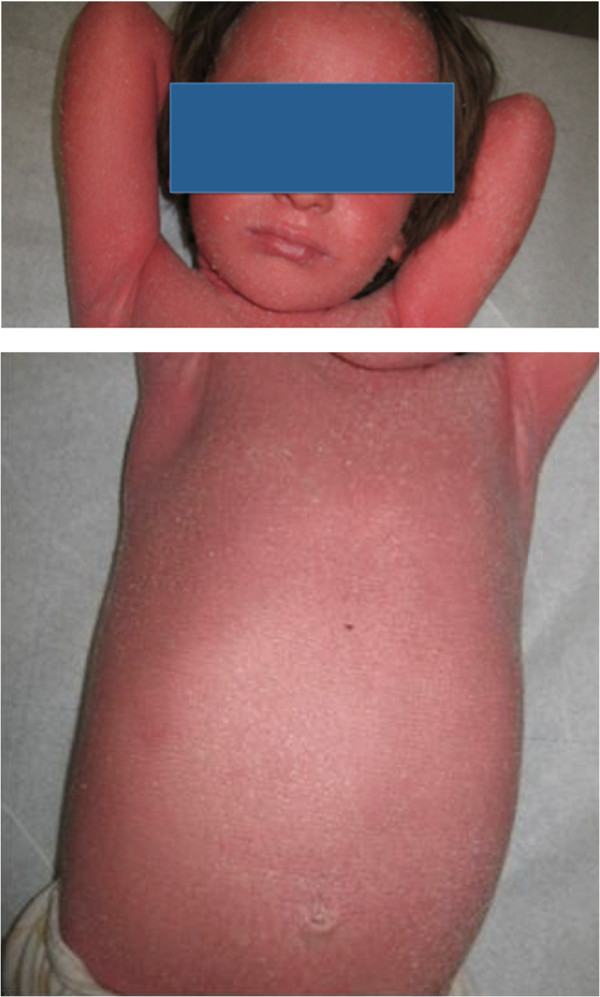
**Clinical pictures of two 3-year-old twins.** Severe pruritic erythroderma involving the entire skin surface with onychodistrophies.

**Figure 2:**
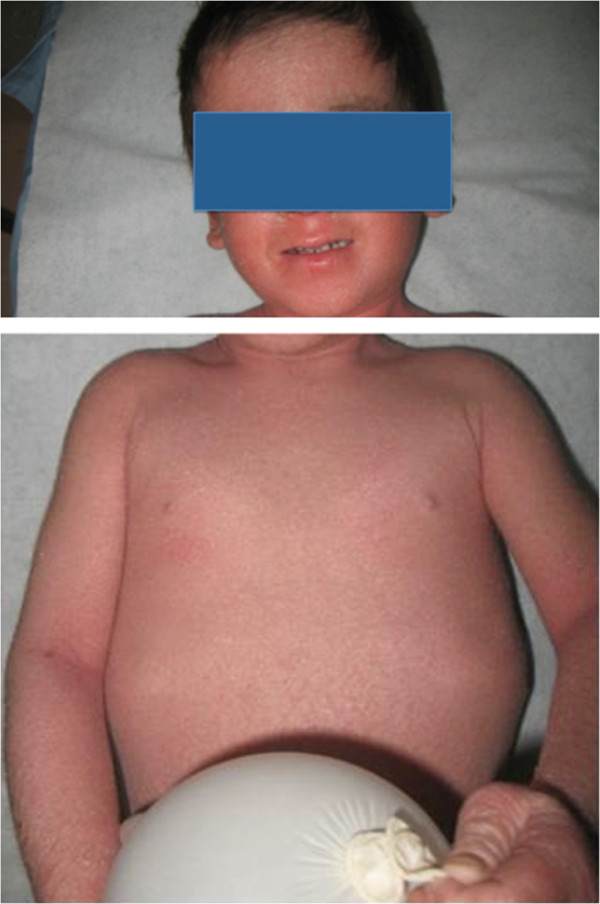
**Clinical pictures of two 3-year-old twins.** Severe pruritic erythroderma involving the entire skin surface with onychodistrophies.

**Figure 3:**
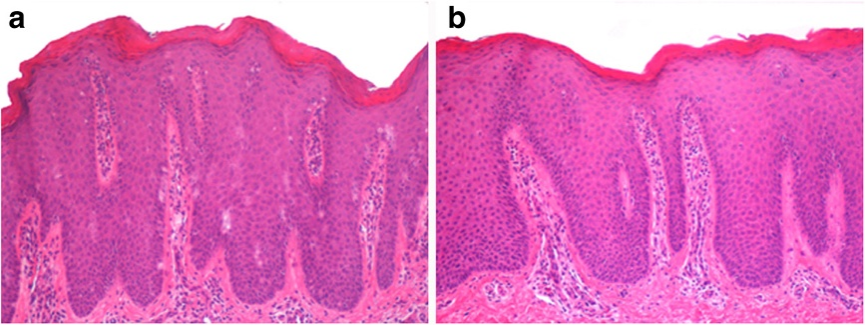
**Microscopic evaluation of Haematoxilyn & Eosin-stained paraffin sections of skin biopsies performed in both twins. a** and **b** Marked elongation of rete ridges, almost absent granular layer and parakeratosis of epidermis associated with inflammatory cells with dilated tortuous vessels in the dermal papillae, consistent with the diagnosis of psoriasis.

**Figure 4:**
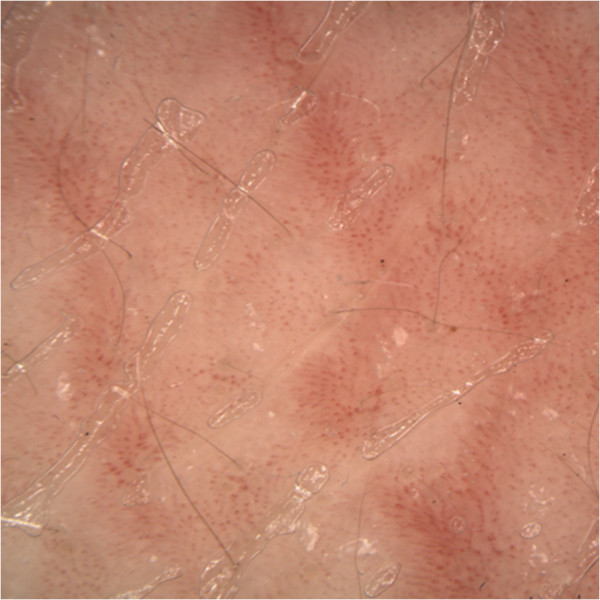
**Dermoscopic features.** Dermoscopy showed bushy glomerular or dotted vessels regularly arranged in a reddish background.

**Figure 5:**
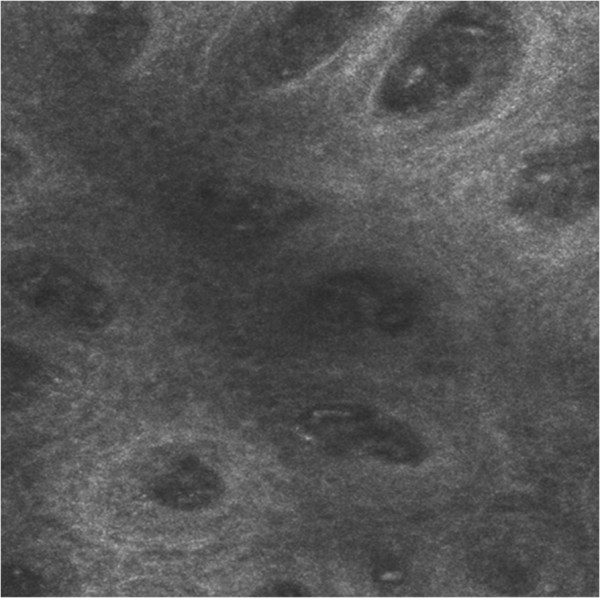
**Confocal reflectance microscopy.** Confocal reflectance microscopy displayed bright nucleated oval cells and dark oval nuclei, described as microabscesses of leucocytes. At dermo-epidermal junction we observed black open rings containing refractive cells.

**Figure 6:**
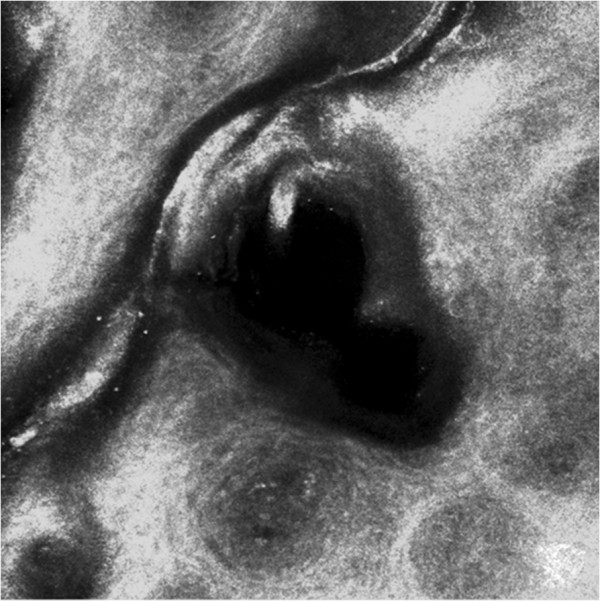
**Confocal reflectance microscopy.** Confocal reflectance microscopy displayed bright nucleated oval cells and dark oval nuclei, described as microabscesses of leucocytes. At dermo-epidermal junction we observed black open rings containing refractive cells.

We visited as consultants two three-year-old Caucasian twins, sister and brother, suffering from an unmanageable erythroderma since the age of 8 months, with extreme distress for parents and pediatricians. At birth, the skin appearance and the health conditions were referred as normal in both newborns, who then had a regular growth. At the time of our visit, a slightly scaling, not exudative, severely pruritic erythroderma was affecting almost the entire skin surface with onychodystrophies in both children, and ectropion in the twin sister (Figures 1, 2). Noteworthy, their father and two other twins on the father’s side also suffer from plaque psoriasis, whereas the father’s twin sister is affected by psoriatic arthritis. Routine blood and culture tests did not show haematological, biochemical, metabolic or infective anomalies. Microscopic evaluation of Haematoxilyn & Eosin-stained paraffin sections [9] of skin biopsies performed in both twins (Figure 3a, b) disclosed marked elongation of rete ridges, almost absent granular layer and parakeratosis of epidermis associated with inflammatory cells with dilated tortuous vessels in the dermal papillae, consistent with the diagnosis of psoriasis. Positive epidermal immunostaining, stronger in the upper layers, for Lympho-Epithelial Kazal-type-related Inhibitor (LEKTI), using polyclonal antibodies D7-12 and D14-16, excluded the diagnosis of Netherton’s syndrome. Furthermore, dermoscopy and confocal reflectance microscopy (RCM) were also considered to validate the diagnosis. Trichoscopy, besides psoriatic hair casts, did not display any hair shaft anomalies as expected in Netherton’s syndrome, whereas cutaneous dermoscopy showed bushy glomerular or dotted vessels, regularly arranged, in a reddish background covered by white scales (Figure 4). RCM displayed, starting from the outer layer, bright nucleated oval cells and dark oval nuclei, described as clusters of polymorphonuclear leucocytes, reduced granular layer and preserved the honeycomb pattern of the stratum spinosum (Figure 5). The dermal papillae were visible as open black and elongated structures, increased in number and diameter. Into the dermal papillary rings, canalicular structures with refractive cells, corresponding to inflammatory cells infiltration, were detectable (Figure 6). Genetic blood investigations revealed Major Histocompatibility Complex, class I, Cw*06 (HLA-Cw*06) expression in both twins and absence of transglutaminase-1 or corneodesmosin or Interleukin (IL)-36 receptor antagonist gene mutations.

**Figure 7:**
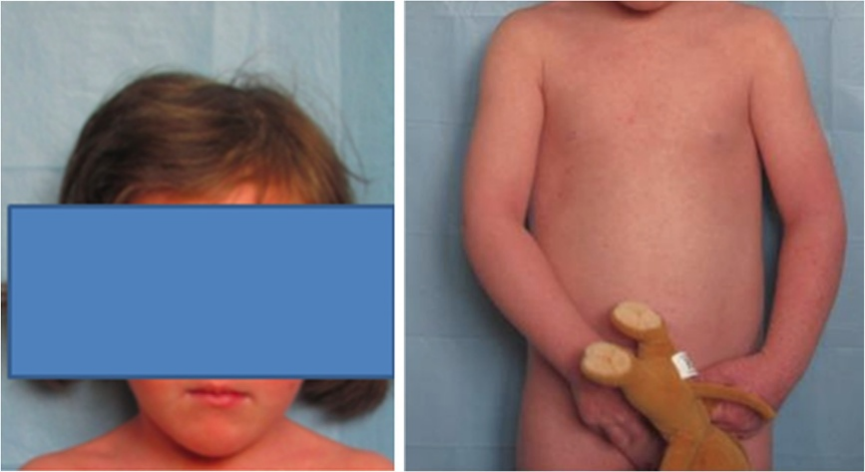
**Clinical images during therapy.** Residual redness of the face and slightly scaling, not infiltrated mild erythematous areas, during treatment.

**Figure 8:**
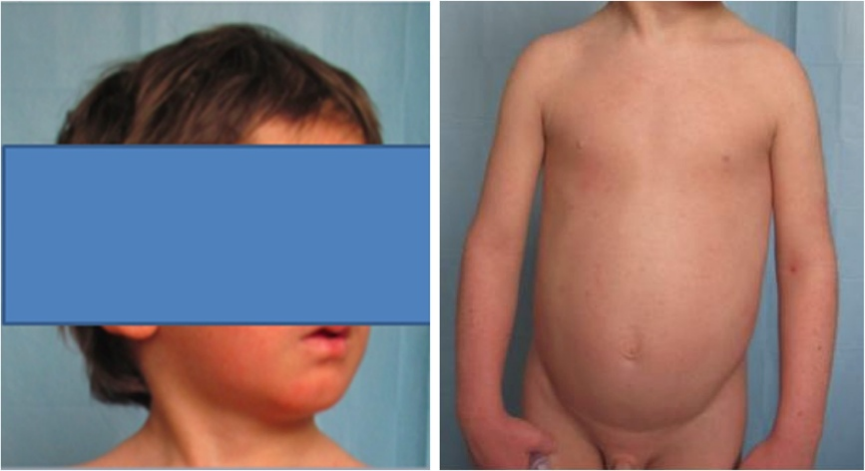
**Clinical images during therapy.** Residual redness of the face and slightly scaling, not infiltrated mild erythematous areas, during treatment.

Taken together, the clinical-pathological features, immunostaining, dermoscopy and RCM findings, genetic HLA-Cw*06 testing, with the high prevalence of psoriasis in the family, were all consistent with the diagnosis of pediatric psoriatic erythroderma. The twins were previously treated with emollients, topical and systemic steroids and short cycles of low-dosage cyclosporine, without any consistent result. Afterwards, etanercept, a tumor necrosis factor-alpha receptor inhibitor approved for psoriatic patients aged 8 years and over, was proposed. After informed consent as off-label treatment because of their young age, etanercept was started at a dosage of 0.8 mg/kg subcutaneously, once a week. After a short intermission because of an upper mild respiratory tract viral infection in both children, etanercept gave ineffective results and was then stopped after a total of 10 injections. Cyclosporine was reconsidered but prescribed at a dosage of 5 mg/kg/day as continuous treatment with close blood monitoring as scheduled. Acitretin, at a dosage of 0.2 mg/kg per day, was combined for one month only, because of the risk of bone anomalies. After one year of continuous treatment, consistent cutaneous clearing and reduction of pruritus with significant improvement of their psychological behaviour were achieved. Weak redness of the face, more evident in the sister, and slightly scaling, not infiltrated mild erythematous areas, without plaque appearance, are still persistent (Figures 7, 8).

### Discussion

Erythroderma present since the age of 8 months in fraternal young children twins is a rare and severe condition difficult to be interpreted and treated. As most of the times, psoriasis is the leading disease among the possible causes of pediatric erythrodermas [1–3]. This report highlights remarkable points: the high prevalence of psoriasis in the family, the intriguing differential diagnoses and the challenging therapeutic options. Familial incidence is higher in early-onset psoriasis, with 37% of adult-onset patients and 49% of pediatric-onset patients having first-degree affected family members [10]. Some studies reported a familial incidence up to 89% in childhood population [11]. The positive upper epidermal immunostaining for protein LEKTI, the absence of transglutaminase-1 or corneodesmosin gene mutations, excluded genodermatoses such as Netherton’ syndrome, autosomal recessive congenital ichthyoses [12] or inflammatory peeling skin syndrome [13], whereas the detection of HLA-Cw*06 in both twins, as susceptibility locus, was strongly consistent with an early-onset psoriasis [14], but the clinical picture and the absence of IL-36 receptor antagonist gene mutations excluded a deficiency of interleukin 36–receptor antagonist also called DITRA syndrome [15]. Furthermore, their personal history, the negative blood and culture tests and the cutaneous histology together with the non-invasive *in vivo* methods of investigations could rule out drug-induced, atopic, infectious or seborrhoeic erythroderma. Pityriasis rubra pilaris, a close differential diagnosis, could be excluded due to the lack of some clinical distinctive features, such as coalescing erythematous perifollicular papules with follicular plugging, islands of pale skin, orange-red scaling erythema, palmoplantar yellowish keratoderma. Furthermore, RCM did not disclose the appearance described in this disorder of keratinization, in particular intermingled foci of parakeratosis and orthokeratosis in the horny layer and around the follicular infundibula, visible as highly refractive nucleated cells including bright polygonal structures and areas of cells with bright borders and dark centers [16, 17]. On the other hand, trichoscopy, dermoscopy and RCM showed typical features of psoriasis. In particular, trichoscopy showed psoriatic hair casts whilst dermoscopy showed bushy glomerular or dotted vessels regularly arranged in a reddish background [18, 19], whereas RCM, starting from the outer layer, displayed bright nucleated oval cells, reduced granular layer, preserved honeycomb pattern, open black and elongated structures into dermal papillary rings and canalicular structures with refractive cells [20, 21]. Altogether, clinical-pathological features, genetic and non-invasive morphological investigations indicated that psoriasis, already remarkably expressed in the father’s family in three fraternal twins, was the cause of the pediatric erythroderma. Once the disease has been diagnosed, the treatment is based on the severity of the skin condition and on the possible presence of joint involvement. Erythroderma, together with widespread, refractory plaques, or generalized pustular psoriasis or psoriatic arthritis, are the most severe expressions of the disease, which commonly require systemic treatment. UV light, systemic retinoids, cyclosporine, methotrexate and etanercept improve the clinical symptoms in childhood psoriasis acting on the main pathways of the psoriatic lesion [22–24]. From their past history, no clinical response was achieved with cyclosporine administered at less than 3 mg/kg. This is why etanercept, the only biologic agent experimented at the time in Europe in childhood and adolescence, was firstly prescribed. Beneficial effects are usually expected already at 4 weeks but, unexpectedly, comparable inconsistent results were noticed in both our young patients. The most common trigger factor in childhood is upper respiratory tract infection, but it is unlikely that the mild transient infective side effect could have had a consistent role in maintaining the disease or in reducing the effectiveness of the drug during etanercept therapy. Cyclosporine, as an immunosuppressant selectively acting on T-cells by calcineurin phosphorylase inhibition, was the most reliable approach to be reconsidered but at higher dosages (5 mg/kg/per day) than those previously experienced as ineffective. The severe skin condition of the two twins led us to combine one-month cycle with oral acitretin - in order to enforce the expected result - but at lower dose to avoid the risk of bone anomalies.

## Conclusions

After one year of continuous cyclosporine treatment we obtained a substantial improvement consisting in cutaneous clearing, significant reduction of pruritus, absence of side effects, and considerable progresses in social and psychological behaviour. In young patients affected by serious skin erythroderma, establishing correct clinical and instrumental procedures is mandatory. This should be supplemented by genetic tests such as those we required to determine the right therapy in terms of cost-benefit and put patients and families in the best position to perform common daily activities.

## Consent

Written informed consent was obtained from the parents of the patients for publication of this Case Report and any accompanying images. A copy of the written consent is available for review by the Editor-in-Chief of this journal.

## Abbreviations

LEKTI: Lympho-Epithelial Kazal-type-related inhibitor
RCM: Confocal reflectance microscopy
HLA-Cw*06: Major histocompatibility complex, class I, Cw*06
IL: Interleukin.
